# Supplementary material for: Experiences of interventions and rehabilitation activities in connection with return-to-work from a gender perspective. A focus group study among employees on sick leave for common mental disorders
Source: PLoS One. 2021 Jun 25;16(6):e0253049. doi: 10.1371/journal.pone.0253049 (PMC8232439; doi:10.1371/journal.pone.0253049)
Supplement: S1 File — (PDF) [file pone.0253049.s001.pdf]

S1 File. Question areas in the interview guide

English translation of the original Swedish version (which can be found further down):

Presentation of the focus group participants

- Could you tell us a little about yourself?
    - Name, home or working conditions, and so on.

Home- and work-related demands and how they affect return to work

- Describe what home- and work-related demands you had during the period before the sick leave.
    - In what way did these demands affect your wellbeing?
- Which home- and work-related demands do you find most difficult to handle?
- How did home- and work-related demands change during your sick leave period?
- In your experience, how do home- and work-related demands affect your return to work?
    - Can you tell us about the home- and work-related demands that constitute the biggest obstacles to return to work?
    - Can you tell us about which home- and work-related resources are supportive for returning to work?

Experiences of the intervention and their effects

- Can you tell us about the help/rehabilitation efforts that you received from the occupational health care and which intervention/interventions you received?
- In your experience, were the rehabilitation efforts useful to facilitate return to work?
    - Can you tell us about what was good and what was less good?
- Did you feel that something was missing but which there was a need for?

- Did any of the rehabilitation efforts affect the home- and work-related demands?

Have the experiences been affected by being a man or a woman

- In your opinion, would any of the experiences that we have spoken of today differed if you had been of the opposite sex? (For example, would the demands at home have looked different? Proposed rehabilitation efforts? Help received from the workplace?)
    - In what way?

Can we contact you later if something comes up in the analyses that we would like to ask additional questions about?

Is there anything that we haven't brought up but that you would like to add? Something that feels unclear or that you are thinking about?

- Give feedback on the discussion.
    - Address points that seemed ambiguous or difficult to understand.
    - Address some key points as a summary.
    - Does this summary agree with what we have talked about?

Probing questions

- Encourage further reasoning with questions such as:
    - What do you mean by that?
    - Can you clarify…?
    - Interesting, what do the rest of you think?
    - How does this relate to… demands/resources/return to work/rehabilitation efforts?

Swedish original version:

Intervjufrågor:

- *Presentation av fokusgruppsdeltagarna*
    - Skulle ni kunna berätta lite om er själva?
        - Namn, hem eller arbetsförhållanden, och så vidare.

- *Hem- och arbetsrelaterade krav och dess påverkan på återgång till arbete*
    - Beskriv hur kraven från arbete och hemmet såg ut tiden innan sjukskrivning.
        - På vilket sätt inverkade dessa krav på hur ni mådde?
    - Vilka hem- och arbetsrelaterade krav upplever ni som svårast att hantera?
    - Hur förändrades hem- och arbetsrelaterade krav under er sjukskrivning?
    - Hur upplever ni att hem- och arbetsrelaterade krav påverkar er återgång i arbete?
        - Kan ni berätta om vilka hem- och arbetsrelaterade krav som utgör de största hindren för att återgå i arbete?
        - Kan ni berätta om vilka hem- och arbetsrelaterade resurser som är stödjande för att återgå i arbete?

- *Upplevelser av interventionen och dess effekter*
    - Kan ni berätta om den hjälp/de rehabiliteringsinsatser som ni fick av företagshälsovården, vilken insats/vilka insatser fick ni?
    - Upplevde ni att rehabiliteringsinsatserna var till nytta för att underlätta återgång i arbete?
        - Kan ni berätta om vad som var bra och mindre bra?
    - Upplevde ni att något saknades, men som det skulle ha funnits ett behov av?

    - Påverkade någon av rehabiliteringsinsatserna hem- och arbetsrelaterade kraven?

- *Ifall man upplever att något av detta påverkats av att vara kvinna eller man*
    - Upplever ni att något av det som vi har pratat om idag hade sett annorlunda ut ifall ni varit av motsatt kön? (T.ex. hade kraven hemma sett annorlunda ut? Föreslagna rehabiliteringsinsatserna? Hjälpen från arbetsplatsen?)
        - På vilket sätt?

- *Är det OK att kontakta er senare ifall det är något som kommer upp i analyserna och som vi önskar att vi hade frågat mera om?*

- *Finns det något som vi missat att ta upp och som ni skulle vilja tillägga? Något som känns oklart eller som blivit kvar i tankarna?*
    - Återkoppla diskussionen.
        - Ta upp punkter som verkade tvetydiga eller svårförståeliga.
        - Ta upp några centrala punkter som summering.
        - Stämmer denna sammanfattning om det som vi har pratat om?

Fördjupande frågor

- Uppmuntra vidare resonemang med frågor typ:
    - Hur menar du med det?
    - Kan du klargöra…?
    - Intressant, vad säger ni andra?

- Hur relaterar detta till… krav/resurser/återgång i arbete/rehabiliteringsinsatserna?
