# Supplementary material for: Experiences of interventions and rehabilitation activities in connection with return-to-work from a gender perspective. A focus group study among employees on sick leave for common mental disorders
Source: PLoS One. 2021 Jun 25;16(6):e0253049. doi: 10.1371/journal.pone.0253049 (PMC8232439; doi:10.1371/journal.pone.0253049)
Supplement: Attachment — Submitted filename: Response to Reviewers.docx [file pone.0253049.s002.docx]

*PONE-D-20-33900*

*Experiences of interventions and rehabilitation activities in connection with return-to-work from a gender perspective. A focus group study among employees on sick leave for common mental disorders.*

We would like to thank the academic editor and reviewers for their time as well as insightful comments and suggestions on how to improve the manuscript. We have responded to the points one-by-one below.

**In response to the points raised by the academic editor:**

1) *Please ensure that your manuscript meets PLOS ONE's style requirements, including those for file naming.*

[*https://journals.plos.org/plosone/s/file?id=wjVg/PLOSOne_formatting_sample_main_body.pdf*](https://journals.plos.org/plosone/s/file?id=wjVg/PLOSOne_formatting_sample_main_body.pdf) *and*

[*https://journals.plos.org/plosone/s/file?id=ba62/PLOSOne_formatting_sample_title_authors_affiliations.pdf*](about:blank)

Thank you for your guidance. We have gone through the manuscript in accordance with the above links and made the following formatting changes:

- The figure and table references have been changed to bold
- The title has been centered on the first page
- The author affiliations have been indicated in superscript
- All references have been given in brackets instead of parenthesis

2) *Please review your reference list to ensure that it is complete and correct. If you have cited papers that have been retracted, please include the rationale for doing so in the manuscript text, or remove these references and replace them with relevant current references. Any changes to the reference list should be mentioned in the rebuttal letter that accompanies your revised manuscript. If you need to cite a retracted article, indicate the article’s retracted status in the References list and also include a citation and full reference for the retraction notice.*

We have ensured that all references are included in the reference list and checked them for accuracy. Some minor edits have been made and one reference that was mentioned twice has now been deleted (and the consequent reference numbers adjusted). We have also gone through the references one-by-one and looked them up on their publishers’ sites but were unable to identify any retracted references. If there are specific reference(s) of concern that you can alert us to, this would be helpful in investigating the matter further.

3. *Please include additional information regarding the interview guide or script used in the study and ensure that you have provided sufficient details that others could replicate the analyses. For instance, if you developed a guide as part of this study and it is not under a copyright more restrictive than CC-BY, please include a copy, in both the original language and English, as Supporting Information*

Thank you. We have now reported in the manuscript that the interview guide was developed within this study (see yellow marking below), and we have also included the guide as Supporting Information in both English and the original, Swedish, language:

“The interview guide included areas that corresponded to the aims of the current and the previous study referred to above (28), such as “Experiences of interventions and their effects”, “Combined demands at home and at work, and how it affects RTW” and “Have the experiences been affected by being a man or a woman”. The interview guide was developed to answer the aims of these studies (see the S1 File for a full account of the included areas and questions). Relevant questions for each area were covered (e.g. “in your experience, did the intervention help you to return to work?”) and (…).”

4.  *We note that you have indicated that data from this study are available upon request. PLOS only allows data to be available upon request if there are legal or ethical restrictions on sharing data publicly. For information on unacceptable data access restrictions, please see* [*http://journals.plos.org/plosone/s/data-availability#loc-unacceptable-data-access-restrictions*](http://journals.plos.org/plosone/s/data-availability#loc-unacceptable-data-access-restrictions)*.*

*In your revised cover letter, please address the following prompts:*

*a) If there are ethical or legal restrictions on sharing a de-identified data set, please explain them in detail (e.g., data contain potentially identifying or sensitive patient information) and who has imposed them (e.g., an ethics committee). Please also provide contact information for a data access committee, ethics committee, or other institutional body to which data requests may be sent.*

*b) If there are no restrictions, please upload the minimal anonymized data set necessary to replicate your study findings as either Supporting Information files or to a stable, public repository and provide us with the relevant URLs, DOIs, or accession numbers. Please see* [*http://www.bmj.com/content/340/bmj.c181.long*](http://www.bmj.com/content/340/bmj.c181.long) *for guidelines on how to de-identify and prepare clinical data for publication. For a list of acceptable repositories, please see* [*http://journals.plos.org/plosone/s/data-availability#loc-recommended-repositories*](http://journals.plos.org/plosone/s/data-availability#loc-recommended-repositories)*.*

*We will update your Data Availability statement on your behalf to reflect the information you provide.*

We have provided the following information in the cover letter on why there are data sharing restrictions:

“Since the data is based on transcribed interviews, the data cannot be completely anonymized and can therefore not be shared openly; although names, places and the like have been removed from the transcripts, the participants speak of situations and sensitive experiences within specific contexts and with a level of detail that would pose a risk for the participants to be recognized. The participants have also not given their consent for the interview transcripts to be freely shared. Requests by certified researchers for access to the data should therefore be made to the Research and Data Office at the Karolinska Institute, via [rdo@ki.se](mailto:rdo@ki.se). If permitted by law and ethical approval, which is decided on a case by case basis, the data can be shared.”

5. *We noted in your submission details that a portion of your manuscript may have been presented or published elsewhere.*

*"Table 1. Participant characteristics is also included in a manuscript pending at BMC Public Health. The same table is used because the sample is the same. However, the two manuscripts address different research questions."*

*Please clarify whether this publication was peer-reviewed and formally published. If this work was previously peer-reviewed and published, in the cover letter please provide* *the reason that this work does not constitute dual publication and should be included in the current manuscript.*

During the current review process, Table 1 has been peer-reviewed and published: Nybergh, L., Bergström, G. & Hellman, T. Do work- and home-related demands and resources differ between women and men during return-to-work? A focus group study among employees with common mental disorders. *BMC Public Health* **20,** 1914 (2020). <https://doi.org/10.1186/s12889-020-10045-4> .

However, the published and the current study have two distinct aims. Whereas the current manuscript explores how employees on sick leave for common mental disorders *experience interventions and rehabilitation activities* during return-to-work, the published article explored how the *experiences of work- and home-related demands as well as resources influence return-to-work* among employees sick-listed for common mental disorders in Sweden. We believe that it is meaningful to present the sample information in Table 1 in both manuscripts as it provides context for the findings. However, if you would prefer that we refer to the other article for details on sample characteristics, we are happy to do so.

Furthermore, the English version of the interview guide (see point 3 above) and a sex-segregated version of Figure 1 (see point 6 below) have also been published in the abovementioned manuscript (the interview guide and the sex-segregated version of the figure were added during the review process of that manuscript). In line with the above, if you would prefer that we refer to the published article for these details, we are happy to do so.

6. *Please upload a copy of Figure 1, to which you refer in your text on line 160. If the figure is no longer to be included as part of the submission please remove all reference to it within the text.*

Thank you for noticing; we have now uploaded the accompanying figure. A sex-segregated version of this figure has also been published in the manuscript mentioned above under point 5, whereas this figure presents the data combined. If you would rather that we republish the same sex-segregated figure from the above-mentioned manuscript, or that we refer to that manuscript directly for these details, we are happy to do so.

**In response to the points raised by the reviewer 1:**

*1. All the participants had received treatment at OHS. I suggest that the authors specify what interventions and what kind of treatment the participants had received.*

Thank you for this relevant comment. The interview guide included a question regarding the type of help and rehabilitation activities that the participants had received to return to work. The following information has now been added to the manuscript:

“All participants had received help from the OHS, which was an inclusion criterion to participate in the study. Previous research on OHS interventions in Sweden has described care as usual to consist of work-directed RTW interventions where usually both the employee and the employer are involved together with an OHS consultant (34). This type of RTW intervention was also mentioned by several of the participants during the interviews. Many also spoke of gradual return to work; workplace adjustments; cognitive-behavioral therapy; stress management, health or mindfulness courses; written or oral information on sleep, workout or nutrition; physical activity ordered by a doctor; visits to a doctor, psychologist, occupational psychologist, counselor or psychiatrist; and medical treatment for depression or sleeping problems. Most participants also mentioned the sick-leave period as an intervention to return to work. A few spoke of group discussion sessions; visits to a physiotherapist or naprapath; referrals to stress clinics; acupuncture; and medical yoga.”

*2. In the current study, the participants emphasized that, “longer initial period of sick leave is seen as important component of successful return”.

Earlier studies have summarized the effective elements of return-to-work include interventions with a focus on work, tailored return-to-work plan, and gradual, early return to work. Furthermore, the results on work modification and partial sick leave have been positive.
The authors take into consideration the earlier finding that sick leave may increase the risk of disability pension (row 631). This might specifically be the case with prolonged sick leave, for which alternatives should be found.
I would suggest, that the authors would discuss their findings related to these findings of earlier studies. e.g.
- Was work modification available for participants?
- How about the possibilities for gradual return or partial sick leave?*

Thank you. We have added the following in the manuscript to address this point (the new parts are marked in yellow), in addition to the previous considerations made on this issue:

“Several participants felt burdened to demonstrate continued ill-health and the need for sick leave during recurring assessments that were made two weeks apart. Some felt this caused them to return to work too early, negatively affecting their RTW in the long run, a finding that is corroborated by other qualitative research (24). By contrast, those who received a long sick leave spell of six weeks at the start felt that it had been an important factor for a successful RTW. However, when it comes to employees sick-listed because of CMD, improved symptoms do not necessarily lead to improved work ability, or vice versa (6), implying that the extent of the sick leave needs to be considered within RTW interventions. Furthermore, work has a positive impact on health and well-being (42), and sick leave increases the risk of future disability pension, recurrent sick leave and unemployment (43). Some studies also indicate that interventions initiated in the first 6 weeks of sickness absence are more favorable to RTW than those initiated later (44). Furthermore, work-focused interventions have been found effectual for partial RTW (6), and a gradual RTW is experienced as an important facilitator to RTW by employees (24). A more flexible and beneficial solution would thus be shorter spells of sick leave with the possibility of regularly assessing the employee’s situation and needs to RTW, including considerations of for example work modification and possibilities for a gradual return, rather than receiving a longer sick leave spell from the beginning. However, while the employees often mentioned work modification and gradual return, they also expressed the need for a clear overview of the RTW-process to reduce feelings of helplessness and confusion in favor of a more focused RTW. Thus, the framing and communication of both the assessments and the extent of the sick leave might warrant attention for it to work in favor of the employee. A scoping review found that positive expectations towards the duration of the sick leave or RTW predicted earlier RTW among employees with CMDs than those who did not have such expectations (45). Considering the employee’s expectations and exploring possibilities to affect them might hence be beneficial to consider during the framing and communication of the sick leave. Additionally, a previous qualitative study on a multidisciplinary RTW program managed by municipal sickness benefit offices found that assessment consultations could create frustration and uncertainty among employees with CMD (25). This is because the employee had difficulties in verbalizing their mental condition to RTW professionals. Our results in an OHS context were similar, but they also show that (…).”

Recited or added references within the revised section above:

- 6. Axén I, Björk Brämberg E, Vaez M, Lundin A, Bergström G, Environmental Health IP. Interventions for common mental disorders in the occupational health service: a systematic review with a narrative synthesis. Int Arch Occup Environ Health. 2020;93:823–838. (Recited)
- 24. Andersen MF, Nielsen KM, Brinkmann S. Meta-synthesis of qualitative research on return to work among employees with common mental disorders. Scand J Work Environ Health. 2012:93-104. (Recited)
- 45. Haitze de Vries AF, Beate Weikert, Alejandra Rodriguez Sanchez & Uta Wegewitz. Determinants of Sickness Absence and Return to Work Among Employees with Common Mental Disorders: A Scoping Review. Journal of Occupational Rehabilitation 2017;28, pages 393–417(2018). (Added)

**In response to the points raised by the reviewer 2:**

*Findings: I would consider consolidating a few of the subsections such as “Individual adaptation at work” and “Organizational interventions” under a broader heading as many elements are work-related – although I’ll leave this up to the authors.*

Thank you for raising this interesting point. We have carefully considered the suggestion and revisited the sub-categories to see if they might overlap. However, we were unable to find an overlap for “individual adaptations at work” and “organizational interventions”. Although both include work-related elements, the content of the former describes adaptations specific to the individual to enhance their worker coping strategies to RTW, whereas the content of the latter depicts experiences at the level of the organization, including work environment and perceived sources of stress. Previous research has also found that the individual and organizational levels as well as their interactions merit separate attention in order to facilitate a sustainable RTW for employees with CMDs (e.g. Nielsen K, Yarker J, Munir F, Bültmann U. IGLOO: an integrated framework for sustainable return to work in workers with common mental disorders. Work Stress. 2018. <https://doi.org/10.1080/02678373.2018.1438536>.)

We also considered if other categories such as “concrete tools to reduce stress” could be combined with some of the other sub-categories, but similarly concluded that they upheld their stability, coherence and distinctiveness from each other, and we have hence kept the sub-categories intact.

*Methodological considerations can be renamed* *Strengths and Limitations.*

Thank you; we have renamed the section accordingly.
